# Pregnancy related factors and temporomandibular disorders evaluated through the diagnostic criteria for temporomandibular disorders (DC/TMD) axis II: a cross sectional study

**DOI:** 10.1186/s12903-024-03930-6

**Published:** 2024-02-13

**Authors:** Giuseppe Minervini, Maria Maddalena Marrapodi, Marco La Verde, Aida Meto, Yuliia Siurkel, Vincenzo Ronsivalle, Marco Cicciù

**Affiliations:** 1 Saveetha Dental College and Hospitals, Saveetha Institute of Medical and Technical Sciences (SIMATS), Saveetha University, Chennai, Tamil Nadu, India; 2 Multidisciplinary Department of Medical-Surgical and Odontostomatological Specialties, University of Campania “Luigi Vanvitelli”, Naples, 80121 Italy; 3 Department of Woman, Child and General and Specialized Surgery, Obstetrics and Gynecology Unit, University of Campania “Luigi Vanvitelli”, Largo Madonna delle Grazie 1, Naples, 80138 Italy; 4 Department of Dentistry, Faculty of Dental Medicine, University of Aldent, Tirana, Albania; 5 International European University School of Medicine, Akademika Hlushkova Ave, 42В, Kyiv, 03187 Ukraine; 6 Department of Biomedical and Surgical and Biomedical Sciences, Catania University, Catania, 95123 Italy; 7 Clinical Microbiology, School of Dentistry, University of Modena and Reggio Emilia, Modena, Italy

**Keywords:** Pregnancy, TMD, Temporomandibular disorders, TMD

## Abstract

**Introduction:**

Temporomandibular disorder (TMD) is a multifaceted condition impacting the chewing system, with its frequency varying across different age groups and showing a higher incidence in women. The involvement of estrogen in TMD has been examined due to the presence of estrogen receptors in the TMJ area. However, the exact effect of estrogen on TMD is complex. During pregnancy, marked by significant hormonal fluctuations, the impact on TMD has been hypothesized but remains unclear due to inconsistent results from various studies.

**Methods:**

In this cross-sectional study, we enrolled 32 pregnant women consecutively. We gathered information on demographics, TMD evaluations (using the Graded Chronic Pain Scale, Jaw Functional Limitation Scale-20, and Oral Behaviors Checklist), and mental health assessments (including Patient Health Questionnaire-9, Patient Health Questionnaire-15, and Generalized Anxiety Disorder-7). We employed descriptive statistics to summarize continuous and categorical data and used t-tests and chi-square tests for comparisons. We also conducted multivariate linear regression, adjusted for demographic factors, to investigate correlations.

**Results:**

The study group mainly consisted of women aged 30–35 (40.6%) and 25–30 (18.8%). Most participants had completed high school (50%) and were married (71.9%). A notable association was found between younger women (under 30) and higher levels of somatic symptoms (*p* = 0.008) and generalized anxiety (*p* = 0.015). Women in their second trimester showed lower severity of somatic symptoms (*p* = 0.04). A significant link was also observed between depression severity and somatic symptom severity (*p* = 0.01). However, we found no significant correlations with other TMD-related health aspects.

**Discussion:**

Our study identified significant associations between psychosomatic and psychological symptoms with variables like age and pregnancy trimester in pregnant women. However, it notably failed to establish a clear relationship between pregnancy-related factors and the severity of temporomandibular disorders (TMD). More comprehensive studies with larger participant pools are necessary to further validate and expand these findings.

## Introduction

Temporomandibular disorder (TMD), encompassing dysfunction in the masticatory system, including the temporomandibular joint (TMJ), muscular and dental components, and supporting bones, is a recognized condition [1]. The occurrence of TMD differs across age groups, with a notably higher prevalence observed among adults, ranging from 40 to 70%. Interestingly, significant TMD incidence has also been noted among individuals in mixed dental dentition [1–7]. Factors contributing to TMD prevalence, causes, and symptoms are influenced by variables such as ethnicity, age, geographical location, and the timing of assessments [ [8–13]. Previous research consistently indicates a higher prevalence of TMD in women, with reported rates being 1.5 to two times more frequent compared to men. This gender difference is attributed to a complex interplay of behavioral, hormonal, anatomical, and psychological factors [12–15]. Furthermore, when considering craniomandibular dysfunctions, women exhibit a fourfold higher susceptibility compared to men [16–26]. Among young women (under 30 years), the prevalence of TMD-related symptoms like clicking jaw, headaches, teeth clenching, reduced jaw mobility, chewing difficulties, and neuromuscular issues is notably elevated [21–34]. Additionally, research demonstrates a significant correlation between symptom severity and age among women, with a relative reduction in clinical manifestations as age advances, a pattern also observed in both sexes [16–19, 21, 22, 35–48].

Estrogen receptors have been identified in TMJ tissues, including the condyle’s chondroid tissue and retrodiscal tissues, suggesting a potential link between estrogen levels and TMD incidence. Estrogen is believed to impact the development, repair, and metabolism of the temporomandibular joint, bone, and related structures [49]. It may influence pain mechanisms associated with TMD as well, given that estrogen receptors are found in both the peripheral and central nervous systems, indicating its capacity to modulate pain signaling [50–52]. Estrogen’s effects on pain perception vary, with physiological pain generally being mitigated by estrogen, while its impact on inflammatory pain is context-dependent. In acute inflammatory pain, estrogen exhibits analgesic properties, whereas in chronic inflammatory pain, it can have a pronociceptive effect due to its presence in both peripheral and central nervous system tissues [53–57].

Hence, the physiological hormonal distinctions between males and females may partly account for the higher TMD prevalence in females. Given the increase in certain female hormones during pregnancy, it is conceivable that pregnant women may experience a higher frequency of TMD-related signs and symptoms compared to non-pregnant women of the same age. However, it is noteworthy to mention that recent studies and meta-analyses have yielded contradictory findings regarding the association between pregnancy and TMD [58–62].

In this article, our focus is on evaluating whether specific pregnancy-related factors exert an influence on the prevalence or severity of TMD.

## Methods

The study adopted a cross-sectional design to investigate the factors affecting TMJ functions among pregnant patients. 32 pregnant women were consecutively enrolled, and at the time of the enrollment demographic information were collected.

**Inclusion criteria**

- Pregnant women at any stage of pregnancy.
- Women aged 18–50 years.
- Willingness to participate in the study and provide informed consent.
- Able to understand and complete the questionnaires in the study language.

**Exclusion criteria**

- Non-pregnant women.
- Patients with a history of TMJ surgery or severe trauma to the TMJ area.
- Women with systemic conditions that might affect TMJ functions (e.g., rheumatoid arthritis, fibromyalgia).
- Inability to understand or complete the questionnaires due to language barriers or cognitive impairments.
- Women with known psychiatric disorders that could interfere with the study assessments.

Various scales and assessments were also used to comprehensively evaluate TMD-related factors and psychological well-being. Here, we provide a detailed description of the data collection process and the scales utilized:

### Data collection

Graded Chronic Pain Scale (GCPS) is a tool used to assess and classify chronic pain in individuals. It was developed by researchers at the University of Washington in Seattle and is designed to provide a more comprehensive understanding of a person’s chronic pain experience beyond just intensity (Table 1) [63]. The CPG scale takes into account several dimensions of chronic pain, including:

**Table 1:** Socio-demographic and clinical characteristics of the sample (*n* = 32)

Item	Description
1	Current pain rating (0–10 scale; 0 = no pain, 10 = pain as bad as it could be)
2	Worst pain intensity in the past 6 months (0–10 scale; 0 = no pain, 10 = pain as bad as it could be)
3	Average pain intensity in the past 6 months (0–10 scale; 0 = no pain, 10 = pain as bad as it could be)
4	Number of days in the last 6 months pain kept you from usual activities (e.g., work, school, housework)
5	Pain interference with daily activities in the past 6 months (0–10 scale; 0 = no interference, 10 = extreme change)
6	Change in ability to participate in recreational, social, and family activities in the past 6 months (0–10 scale; 0 = no change, 10 = extreme change)
7	Change in ability to work (including housework) due to pain in the past 6 months (0–10 scale; 0 = no change, 10 = extreme change)

Pain Intensity: This dimension assesses the severity of pain on a scale from 0 to 10, with 0 being no pain and 10 being the worst pain imaginable.

Pain-Related Disability: This dimension evaluates how much chronic pain interferes with a person’s daily activities, including work, social life, and self-care.

Days in Pain: This dimension assesses how many days in the past six months a person has experienced significant pain.

Pain Intensity Variability: It considers whether the pain is relatively constant or if it fluctuates over time. Based on the scores in these dimensions, individuals can be categorized into one of five grades, ranging from Grade 0 to Grade IV.

JPLS-20: The Jaw Functional Limitation Scale-20 subscales, including Mastication, Mobility, Communication, and Global, provide a comprehensive assessment of TMJ-related function and overall health status.

Oral Behaviors Checklist (OBC): It is a tool used in the field of speech-language pathology and dentistry to assess and document oral behaviors in individuals, particularly in children. It is often used to evaluate oral habits and behaviors that may impact speech, swallowing, and dental health. The OBC is typically completed through observation and may involve input from parents, caregivers, or teachers who are familiar with the individual’s behaviors. The Oral Behaviors Checklist typically includes a list of behaviors related to oral function and habits.

Psychological well-being was a central aspect of the study, and it was thoroughly assessed using standardized measures. Specifically, three widely recognized assessment tools were employed to gauge different dimensions of psychological well-being: The Patient Health Questionnaire-9 (PHQ-9), the Patient Health Questionnaire-15 (PHQ-15), and the Generalized Anxiety Disorder-7 (GAD-7) scale.

The PHQ-9 is a self-report questionnaire designed to evaluate the severity of depression symptoms in individuals. It consists of nine items, each corresponding to a specific symptom of depression. Participants are asked to rate the frequency of these symptoms over the past two weeks on a scale ranging from 0 (not at all) to 3 (nearly every day). The items cover a broad spectrum of depressive symptoms, including low mood, anhedonia (loss of interest or pleasure), changes in sleep patterns, changes in appetite or weight, feelings of worthlessness or guilt, difficulty concentrating, fatigue, psychomotor agitation or retardation, and thoughts of self-harm or suicide. Total scores on the PHQ-9 can range from 0 to 27, with higher scores indicating greater depression severity. Typically, participants with scores of 5–9 are considered to have mild depression, 10–14 moderate depression, 15–19 moderately severe depression, and 20 or more severe depression.

The PHQ-15 is another self-report questionnaire used to assess somatic symptom severity, which is an essential component of overall psychological well-being. The PHQ-15 includes 15 items that inquire about the presence and severity of various physical symptoms, such as headaches, stomach pain, back pain, and fatigue, over the past four weeks. Participants rate the symptoms on a scale ranging from 0 (not bothered at all) to 2 (bothered a lot). The total score on the PHQ-15 can range from 0 to 30, with higher scores indicating a greater burden of somatic symptoms. This assessment helps to identify individuals with somatic symptom disorders and provides valuable insights into the physical manifestations of psychological distress.

The GAD-7 is a self-report questionnaire designed to measure the severity of generalized anxiety disorder (GAD) symptoms in individuals. It comprises seven items that inquire about common anxiety symptoms, such as excessive worrying, restlessness, difficulty controlling worry, muscle tension, sleep disturbances, and irritability, over the past two weeks. Participants rate the frequency of these symptoms on a scale ranging from 0 (not at all) to 3 (nearly every day). The total score on the GAD-7 can range from 0 to 21, with higher scores indicating greater anxiety severity. Similar to the PHQ-9, participants with scores of 5–9 are typically considered to have mild anxiety, 10–14 moderate anxiety, and 15 or more severe anxiety.

These standardized assessment tools, including the PHQ-9, PHQ-15, and GAD-7, allow for a comprehensive evaluation of participants’ psychological well-being, covering domains of depression, somatic symptomatology, and generalized anxiety. The use of such validated instruments ensures the reliability and validity of the psychological assessments in the study. The questionnaires were collected between March and December 2022. The data of the questionnaires were digitalized and analyzed at the Department of Dentistry, Faculty of Dental Sciences, ALDENT UNIVERSITY and this study was approved by the institute’s ethical committee [Protocol no. 846/2022; Date: 05/05/2022].

### Statistical analysis

Descriptive statistics were utilized to summarize the study population. For continuous variables such as pain severity and functional scores, population characteristics were described using means and standard deviations (SD). For categorical variables such as the presence of oral behaviors or specific demographic characteristics, numbers and percentages were calculated to provide a clear overview of the study population.

Univariate regression analyses were employed to explore the associations between various pregnant woman demographic and clinical characteristics and the aforementioned scales, including pain perception, chronic pain grade, JFLS functional scores, OBC scores, and psychological well-being measures. These analyses aimed to identify potential relationships between individual factors and TMD functions.

Subsequently, multivariate regression analyses were conducted to further evaluate these associations, considering a range of independent variables, including pregnancy trimester, BMI, comorbidity, smoking status, age, education, marital status, and psychological well-being scores. The multivariate analyses allowed for a more comprehensive understanding of the combined effects of these variables on TMD functions among pregnant women.

Overall, the study design, data collection process, and statistical analyses were meticulously conducted to provide a detailed examination of the factors influencing TMD functions in pregnant patients, shedding light on the complex interplay between pregnancy status, demographics, and psychological well-being.

### Sample size

The calculated sample size for this single population study with an anticipated mean of 20, a known population standard deviation of ± 10, an alpha (α) level of 0.05, and a desired statistical power of 90%, the determined sample size is 27.

## Results

### Patient characteristics

Table 2 offers a concise overview of their socio-demographic and clinical profiles. Maternal age exhibited heterogeneity, with the majority falling within the age brackets of 30 to 35 years (40.6%) and 25 to 30 years (18.8%). The average pre-pregnancy Body Mass Index (BMI) was 25.68 ± 4.53 kg/m². Educational backgrounds varied, with a significant proportion having completed high school (50%) and a quarter having attained secondary school education (25%). A subset of the cohort reported tobacco use (18.8%). Pregnancy-related comorbidities included gestational diabetes (6.3%) and gestational hypertension (3.1%), while no instances of preeclampsia were observed. A majority of conceptions were spontaneous (84.4%), and marital status showed that 71.9% of participants were married, while 28.1% were single or celibate. The racial composition predominantly comprised individuals of White ethnicity (96.9%), with a smaller representation of Black individuals (3.1%). This diverse sample offers valuable insights into the characteristics of the population under investigation.

**Table 2:** Socio-demographic and clinical characteristics of the sample (*n* = 32)

	Total sample (n 32)
Maternal age, years (%)	
18-20 y	1 (3.1)
20-25	3 (9.4)
25-30	6 (18.8)
30-35	13 (40.6)
35-40	6 (18.8)
40-45	2 (6.3)
45-50	1 (3.1)
BMI prior to pregnancy, kg/m^2^	
Mean ± SD	25.68 ± 4.53
Level of education, N (%)	
Primary School	0 (0.0)
Secondary School	8 (25.0)
High School	16 (50.0)
Degree	8 (25.0)
Tobacco use, yes, N (%)	6 (18.8)
Comorbidities, N (%)	
Gestational diabetes	2 (6.3)
Gestational hypertension	1 (3.1)
Preeclampsia	0 (0.0)
Conception, N (%)	
Spontaneous	27 (84.4)
In-vitro fertilization	5 (15.6)
Marriage, N (%)	23 (71.9)
Celibacy, N (%)	9 (28.1)
Race, N (%)	
White	31 (96.9)
Black	1 (3.1)

**Fig. 1:**
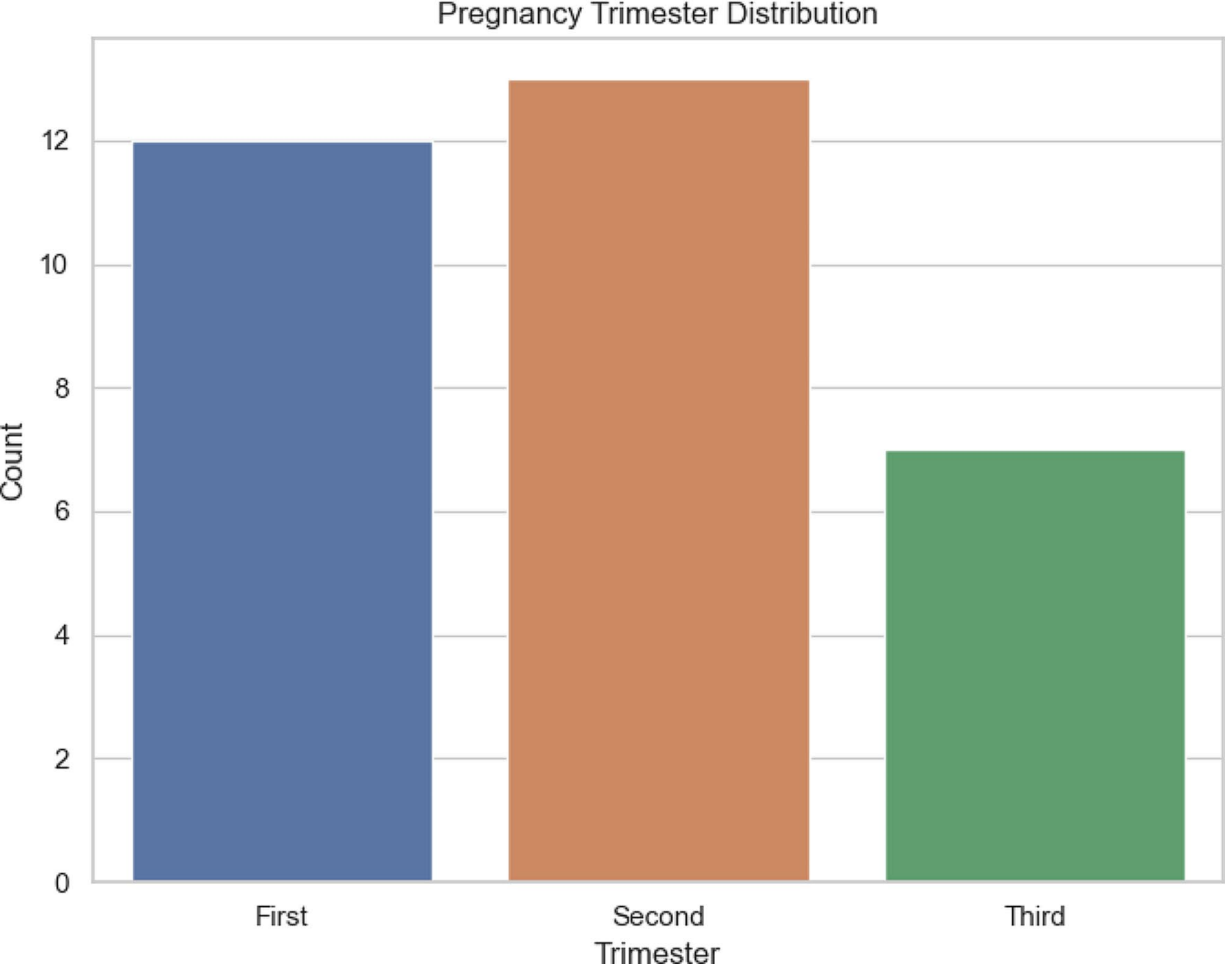
This figure illustrates the distribution of the number of subjects within each trimester group during pregnancy, offering an overview of the sample distribution across different pregnancy stages

In Fig. 1 is represented the number of women for each trimester: 12 in the first trimester, 13 in the second trimester and 7 in the third one.

### Association between subject features and health-related measures relevant to TMD functions

To comprehensively analyze the intricate interplay between pregnancy status, demographics, and psychological variables, we employed both univariate and multivariate regression analyses.

In our initial univariate analyses, we assessed the relationships between several demographic and clinical factors and various health-related measures relevant to TMD functions.

Initially, we explored the potential impact of Body Mass Index (BMI), considering its relevance during pregnancy. However, our findings indicated that BMI did not serve as a significant predictor for TMD-related health outcomes within our sample of 32 pregnant women.

Looking at age, younger ages (< 30 yo) exhibited a strong association with the presence of somatic symptom severity (*p* = 0.008) and generalized anxiety (*p* = 0.015) among pregnant women. However, it did not significantly predict other health-related factors associated with TMD functions.

**Fig. 2:**
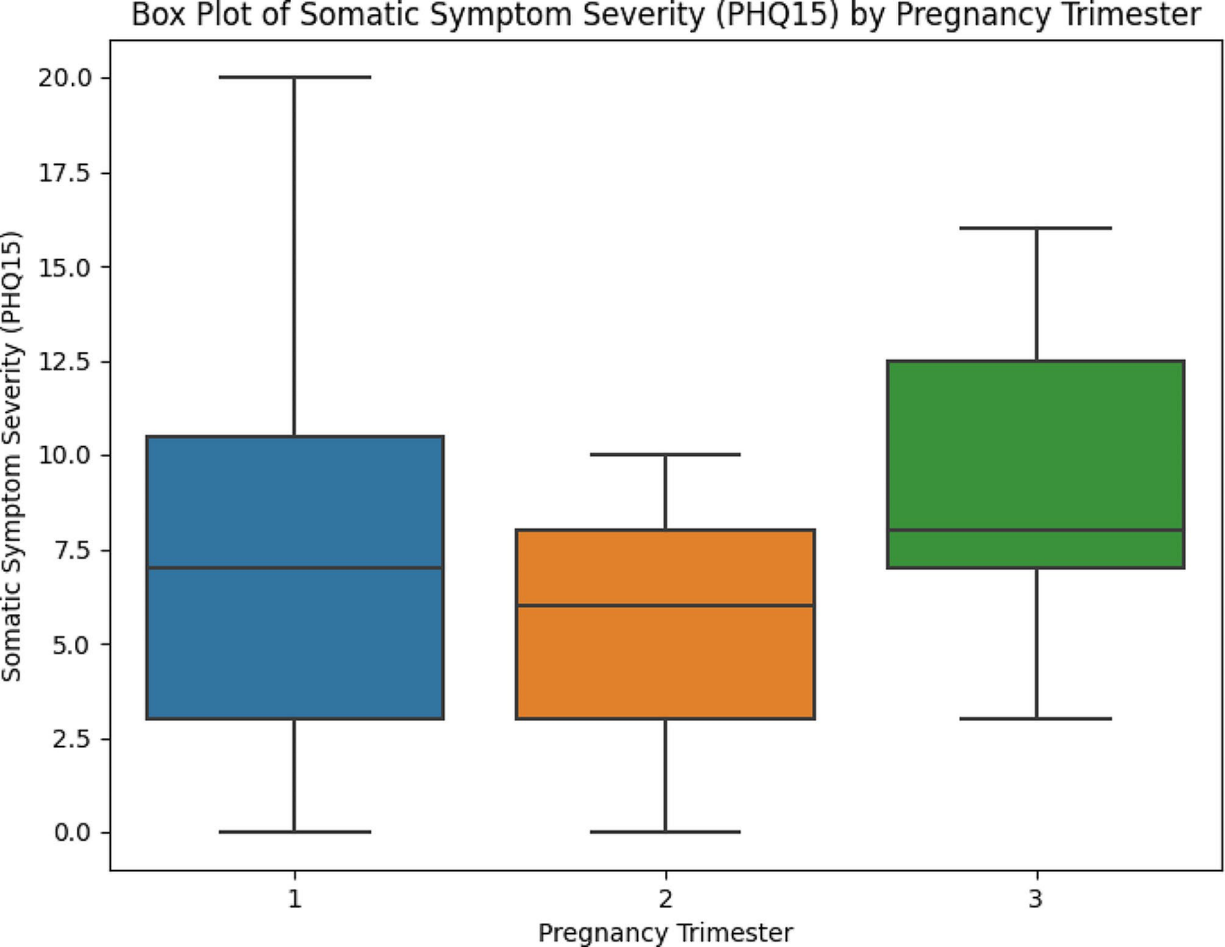
This figure presents the mean value of “Somatic Symptom Severity (PHQ15)” stratified by trimester groups during pregnancy. It provides insights into the variation in somatic symptom severity across different stages of pregnancy

Furthermore, we explored the predictive power of marital status and education. Marital status significantly predicted somatic symptom severity but did not significantly influence other health-related factors relevant to TMD functions (Fig. 2).

Education, on the other hand, did not emerge as a significant predictor for any of the examined health-related outcomes.

To provide a comprehensive understanding of the factors influencing TMD functions in pregnant women, we conducted multivariate regression analyses. These analyses encompassed pregnancy trimester, along with other relevant demographic and psychological variables, as independent variables. Our multivariate analysis revealed that the second trimester of pregnancy was significantly associated with lower somatic symptom severity scores related to TMD compared to the first trimester (*p* = 0.04). Additionally, higher scores on the Patient Health Questionnaire-9 (PHQ9), reflecting depression severity, were associated with increased somatic symptom severity scores (*p* = 0.01). Regarding orofacial parafunction, pregnancy trimester, BMI, comorbidity, and PHQ9 did not exhibit statistically significant relationships within our pregnant population.

Generalized anxiety, as measured by the Generalized Anxiety Disorder-7 (GAD7), did not show significant associations with any of the independent variables among pregnant women. Similarly, depression severity, as assessed by PHQ9, did not significantly relate to the examined independent variables among our pregnant study participants. Furthermore, when assessing other health-related outcomes related to TMD functions, such as global functioning, communication, mobility, mastication, chronic pain grade, characteristic pain intensity, and the number of body areas with pain, none of these dependent variables demonstrated statistically significant relationships with the independent variables considered in our regression analyses.

## Discussion

The findings of this study provide valuable insights into the complex interplay of factors influencing TMD functions in pregnant women. TMD is a multifaceted condition that affects various components of the masticatory system, including the TMJ, muscles, and supporting structures. It is characterized by a range of symptoms, such as jaw pain, clicking, headaches, and reduced jaw mobility. This study aimed to explore the impact of pregnancy-related factors, demographics, and psychological well-being on TMD functions among pregnant women [1–3].

One of the intriguing aspects of TMD is its differential prevalence across age groups. The study’s findings corroborate previous research indicating that TMD is more prevalent among adults, with rates ranging from 40 to 70%. This underscores the importance of understanding TMD in a broader demographic context and suggests that interventions and preventive measures may need to be tailored to different age groups.

Gender disparities in TMD prevalence have long been recognized, with women being disproportionately affected. The study’s results support this observation, revealing that women are 1.5 to two times more likely to experience TMD compared to men. Furthermore, when considering craniomandibular dysfunctions, this gender difference becomes even more pronounced, with women exhibiting a fourfold higher susceptibility. Among young women under 30 years of age, the prevalence of TMD-related symptoms is notably elevated. This emphasizes the need for targeted interventions and increased awareness among healthcare providers, especially when treating female patients, to address the unique challenges faced by this population [12, 13, 59].

Estrogen, a key hormone, has been implicated in TMD. Estrogen receptors identified in TMJ tissues suggest a potential link between estrogen levels and TMD incidence. The study’s findings align with this hypothesis, emphasizing the hormonal distinctions between males and females as a contributing factor to the higher TMD prevalence in females. The role of estrogen in modulating pain mechanisms associated with TMD is particularly intriguing. While estrogen has been shown to mitigate physiological pain, its effects on inflammatory pain are context-dependent. This highlights the intricate relationship between hormones and pain perception, underscoring the need for further research into the mechanisms underlying TMD in the context of hormonal fluctuations [54–57, 64–69].

Pregnancy is a period characterized by significant hormonal changes, including increased levels of certain female hormones. This study sought to explore whether pregnant women might experience a higher frequency of TMD-related signs and symptoms compared to their non-pregnant counterparts. The findings, however, do not conclusively support this hypothesis. Recent studies and meta-analyses have yielded contradictory results regarding the association between pregnancy and TMD. While it was reasonable to speculate that hormonal changes during pregnancy might influence TMD, this study did not find a significant link between pregnancy status and TMD functions [58, 70, 71].

The study’s detailed methodology, including the use of standardized assessments and careful data collection, contributes to the understanding of TMD in pregnant women. Pain perception, chronic pain grade, functional status, oral behaviors, and psychological well-being were comprehensively evaluated. The PHQ-9, PHQ-15, and GAD-7 scale were employed to assess different dimensions of psychological well-being, including depression, somatic symptom severity, and generalized anxiety.

The results of the multivariate regression analyses shed further light on the factors influencing TMD functions during pregnancy. The second trimester of pregnancy was found to be significantly associated with lower somatic symptom severity scores related to TMD compared to the first trimester. This suggests that the timing of pregnancy may influence the manifestation of TMD-related symptoms, with potential implications for clinical management. Additionally, higher depression severity scores, as measured by the PHQ-9, were linked to increased somatic symptom severity scores, hinting at a possible connection between depression and TMD functions during pregnancy. This highlights the importance of addressing psychological well-being as part of TMD management among pregnant women.

However, it is important to note that not all health-related measures relevant to TMD functions exhibited significant relationships with the examined independent variables. Factors such as BMI, smoking status, education, and other health-related outcomes did not show significant associations within the pregnant population. These findings underscore the complexity of TMD and the need for further research to unravel the intricacies of its etiology and management, especially in the unique context of pregnancy [59, 72].

Temporomandibular disorders (TMD) represent a significant health concern that impacts various aspects of life, including pain perception, psychological well-being, and quality of life. The research articles by Boening et al., Seweryn et al., and Wieckiewicz et al. provide valuable insights into these aspects [73–76].

Boening et al. emphasized the complex nature of TMD, highlighting that it encompasses symptoms related to the dysfunction of the temporomandibular joints and associated muscles. Symptoms can range from pain and tenderness in the TMJ area to headaches, earaches, and even tinnitus. The multifactorial etiology of TMD, including psychological, occlusal, and general health factors, necessitates a multidisciplinary approach for effective diagnosis and treatment. This perspective is essential for understanding TMD in women, who often report higher instances of TMD-related symptoms, potentially due to hormonal influences, stress, and other psychosocial factors.

Seweryn et al. conducted a study focusing on the relationship between TMD, life satisfaction, and sleep quality among Polish adults. They found that TMD patients with average life satisfaction levels had significantly higher levels of muscle pain. Moreover, a large proportion of these patients experienced poor sleep quality, which was statistically associated with higher levels of muscle pain. Interestingly, women in this study reported worse sleep quality than men, pointing to gender-specific differences in the impact of TMD.

Wieckiewicz et al. explored the psychosocial dimensions of TMD, assessing pain intensity, disability, anxiety, depression, and stress in Polish adults with TMD. Their findings revealed a high prevalence of stress, anxiety, and depression among TMD patients, with females displaying more depression symptoms. The study underscored the significant correlation between masticatory muscle disorders and depression, stress, pain intensity, and disability. These findings highlight the critical need for an interdisciplinary approach in managing TMD, considering both physical and psychosocial aspects.

In summary, these studies collectively illustrate that TMD is not just a physical ailment but is intricately connected to psychological and social factors. This understanding is particularly relevant for women, who may experience TMD differently due to various biological and psychosocial influences. The need for a multidisciplinary approach in diagnosis and treatment, considering both the physical symptoms and the psychosocial impacts, is crucial for effective management of TMD in the general population, with particular attention to the unique needs of women.

In discussing the connection between oral health and pregnancy, Radwan-Oczko et al. study is particularly enlightening. Their research aimed to assess pregnant women’s self-awareness regarding oral health and its importance during pregnancy. The study found that a significant portion of women (24%) lacked awareness of the importance of proper oral hygiene during pregnancy. Furthermore, only 20% had undergone an oral examination before pregnancy, and 38.5% did so after confirming their pregnancy. A noteworthy 41.5% reported dental problems during pregnancy, and 30.5% received dental treatment. The study highlighted that better knowledge about oral health during pregnancy was associated with higher education and living in big cities. Interestingly, there was a significant correlation between higher birth weight and more frequent tooth brushing. This research underscores the need for enhanced education about oral health in pregnancy, as many women are insufficiently informed about its significance for both their health and the development of their fetus. This study has several limitations. First, the reliance on self-reported questionnaires may lead to subjective bias, as these measures depend on participants’ perceptions and recollections, which can be influenced by individual differences and current mood states. Secondly, the small sample size of 32 pregnant women limits the generalizability of our findings to a broader population. Additionally, the absence of objective diagnostic measures and a control group restricts our ability to draw firmer conclusions about the causality and extent of TMD in pregnant women. These limitations highlight the need for further research with larger, more diverse samples and a mix of subjective and objective methodologies to deepen our understanding of TMD during pregnancy.

In conclusion, our study identified significant associations between psychosomatic and psychological symptoms with variables like age and pregnancy trimester in pregnant women. The findings emphasize the importance of considering the timing of pregnancy and addressing psychological well-being when assessing and managing TMD in pregnant patients. However, further research with larger sample sizes is needed to confirm and expand upon these findings, ultimately enhancing our ability to provide effective care for individuals experiencing TMD during pregnancy.

## Data Availability

The corresponding author will have access to the data that were the basis for this article.
